# Biocompatibility and Connectivity of Semiconductor Nanostructures for Cardiac Tissue Engineering Applications

**DOI:** 10.3390/bioengineering9110621

**Published:** 2022-10-27

**Authors:** Roberto Gaetani, Yuriy Derevyanchuk, Andrea Notargiacomo, Marialilia Pea, Massimiliano Renzi, Elisa Messina, Fabrizio Palma

**Affiliations:** 1 Department of Molecular Medicine, “Sapienza” University of Rome, 00176 Rome, Italy; 2 Institute for Photonics and Nanotechnologies (IFN), National Research Council of Italy, 00133 Rome, Italy; 3 Department of Physiology and Pharmacology, “Sapienza” University of Rome, 00176 Rome, Italy; 4 Policlinico Umberto I, “Sapienza” University of Rome, 00176 Rome, Italy; 5 Department Ingegneria dell’ Informazione, Elettronica e Telecomunicazioni, “Sapienza” University of Rome, 00176 Rome, Italy

**Keywords:** biocompatibility, electronic sensing, zinc oxide nanowires, silicon nanowires, cardiac tissue

## Abstract

Nano- or microdevices, enabling simultaneous, long-term, multisite, cellular recording and stimulation from many excitable cells, are expected to make a strategic turn in basic and applied cardiology (particularly tissue engineering) and neuroscience. We propose an innovative approach aiming to elicit bioelectrical information from the cell membrane using an integrated circuit (IC) bearing a coating of nanowires on the chip surface. Nanowires grow directly on the backend of the ICs, thus allowing on-site amplification of bioelectric signals with uniform and controlled morphology and growth of the NWs on templates. To implement this technology, we evaluated the biocompatibility of silicon and zinc oxide nanowires (NWs), used as a seeding substrate for cells in culture, on two different primary cell lines. Human cardiac stromal cells were used to evaluate the effects of ZnO NWs of different lengths on cell behavior, morphology and growth, while BV-2 microglial-like cells and GH4-C1 neuroendocrine-like cell lines were used to evaluate cell membrane–NW interaction and contact when cultured on Si NWs. As the optimization of the contact between integrated microelectronics circuits and cellular membranes represents a long-standing issue, our technological approach may lay the basis for a new era of devices exploiting the microelectronics’ sensitivity and “smartness” to both improve investigation of biological systems and to develop suitable NW-based systems available for tissue engineering and regenerative medicine.

## 1. Introduction

Monitoring and conditioning ionic currents and potentials across cell membranes is key in various fields, from basic cardiology and neuroscience to precision pharmacology, bio-medical device engineering and robotics in medicine.

In the cardiac field, the use of induced pluripotent stem cells (iPSCs) has allowed the development of new in vitro “tissue models” that can mimic both healthy and pathological diseases [1,2]. Such cells can be isolated from different patients and differentiated into cardiomyocytes, cultured together with supporting cells such as cardiac stromal or cardiac endothelial cells to generate in vitro 3D tissues that can be used for both in vitro or in vivo applications [3]. In this context, the integration of electronic devices for electrical tissue stimulation has been proven advantageous in term of tissue maturation and functional assembly of cardiomyocytes in vitro [4,5,6,7]. Similarly, the development of new semiconductive biomaterials in combination with traditional biomaterials to improve the conductivity of the composite materials and subsequent tissue maturation has been also investigated [8,9,10]. Given the electrical proprieties of excitable cell membranes (neurons; cardiomyocytes; muscular cells), the understanding of the relationship between their functional connectivity with their physiological or pathological features has represented another goal not only to improve our knowledge in cardiac biology and neurosciences but also to design new devices for tissue engineering applications as well as for drug screening and disease modeling.

One of the major goals in cardiac tissue engineering is the realization of an electronic device devoted to electrophysiological recording from, possibly, a large network of cells [11]. Recently, vertical nanopillar electrodes have been used to record action potentials from cardiomyocytes in culture [12]. Of note, thanks to the reversible, nanoscale electroporation of the membrane attainable by nanopillars, action potentials could be recorded both extracellularly and intracellularly from these cells [13]; the pharmacological modulation of action potentials related to the application of ion channel drugs could also be investigated [12]. Furthermore, as mentioned previously, the recent development in 3D tissue models has also raised the attention of biotechnology to couple nanowire (NW)-based scaffold-rich and scaffold-free systems for cardiac microtissue differentiation and electromechanical coupling [8,9]. A particular effort to move forward is now focusing on the attempt to substitute passive electrodes with nanometric active electrodes, such as NW transistors [14,15] or nanotube-coupled transistors [16]. The goal is naturally to combine a suitable sensing technique with the large-scale integration typical of microelectronics. This achievement would be of greatest importance for both scientific and commercial reasons: a microelectronic sensing device would allow unparalleled description of the cell electrical activity and signal propagation, thus providing a higher level of electrical mapping of the spike activity, from complex networks to single cells and within cellular sub-domains. Such an instrument would prove crucial for both basic and translational research as well as for large-scale, pharmacological screening purposes [17].

To date, such a combination could not be obtained, mainly due to two reasons: (i) the difficulty of achieving a suitable electric contact between the detector and the cell membrane, and (ii) the difficulty of growing small-sized nanostructures compatibly with the standard CMOS technology. Therefore, microelectromechanical systems (MEMS) technology can produce pillars and wires, but these cannot reach the suggested dimension of about 20 nm. Other nanoscale fabrication technologies, such as electron beam lithography can be, in principle, extended to large wafers, but are costly and have low throughput.

Here, we evaluated the effects of NWs produced by two different technologies, namely hydrothermal synthesis of ZnO NWs and VLS growth of silicon NWs, both working at low temperature and, thus, capable of safely growing nanowires on the backend of the IC, on primary cardiac stromal cells (CSCs). In our work, we evaluated how the degradation of Si and ZnO NWs can occur in the presence of biological fluids such as cell culture media as well as how the length of the NWs can affect such degradation and, thus, potential NW cytotoxicity. Moreover, we evaluated for the first time CSCs’ survival and phenotype when cultured on top of Si and ZnO NWs and also whether the NWs’ length can affect such behavior. These cells have been shown to play a key role in tissue homeostasis. They support ECM maintenance and angiogenesis and exert many paracrine functions for the benefit of cardiomyocytes, vessels and immune cells and, therefore, are fundamental in the development of multicellular 3D cardiac tissue models [18,19,20].

## 2. Materials and Methods

### 2.1. ZnO Nanowire and Si Nanowire Synthesis

For the ZnO nanowire technology adopted here, an ultrathin (few-nanometer thick) ZnO film (“seed layer”) was preliminarily deposited on (001) oriented Si wafer chips by the reactive sputtering technique as previously described [21]. The wet chemical growth of NWs then took place at a temperature as low as 80 °C by hydrothermal synthesis in an equimolar (10 mM) aqueous solution of zinc nitrate hexahydrate (Zn(NO_3_)_2_·6H_2_O) and hexamethylenetetramine (HMTA) using ultrapure water, for 1 and 3.5 h, respectively. After growth, the NWs samples were rinsed in isopropyl alcohol and soft-dried in ultrapure nitrogen flow. To highlight the surface morphology and evaluate the difference in NW length, 3 samples were analyzed by field-emission electron scanning microscopy. The evaluation of the NWs’ length was made by cross-sectional SEM imaging of sample borders cleaved across the NWs’ growth surface. Images were collected at 45° stage tilt; then, the measured lengths were corrected for the tilt view angle. For each sample, three images were collected in different sites and analyzed, and a total of 30 measurements were used for the evaluation of average length. Methods for the preparation of Si nanowires have been thoroughly described previously [22].

### 2.2. Cell Culture

Cardiac stromal cells (CSCs) were isolated from human heart biopsies as previously described [23] and kept in culture in Iscove’s modified Dulbecco’s medium supplemented with 20% FBS, penicillin–streptomycin, and L-glutamine at 37 °C and 5% CO_2_. Briefly, cardiac tissue was minced into small pieces and cultured on fibronectin-coated dishes. After 2–3 weeks, outgrowth cells were collected, expanded and used for the experiments (passage 6–10).

### 2.3. AlamarBlue Metabolic Activity Assay

To evaluate the effects of Si and ZnO NWs on cell survival, growth and morphological modifications, 40 × 10^3^ per cm^2^ primary cardiac stromal cells (CSCs) were cultured on top of ZnO NWs grown for either 1 (ZnO-NW_1h) or 3.5 h (ZnO-NW_3.5h) on top of ZnO-coated Si wafer chips or Si NWs for up to 10 days. An ultrathin (few-nanometer thick) ZnO film deposited on Si (ZnO wafer), a Si wafer or regular tissue culture plastic (TC) was used as the control seeding substrate. The metabolic activity of CSCs in culture was evaluated using an AlamarBlue metabolic activity assay, which uses the reducing environment of the living cells to assess cell viability and number. In the presence of cells, resazurin, the active component of AlamarBlue, is reduced, and the change from the oxidized blue state to the reduced pink state is indicative of cell number and viability and can be detected either by absorbance or fluorescence. Briefly, AlamarBlue was diluted 1:10 in cell culture media 10% *v*/*v*; Invitrogen, Waltham, MA, USA), n = 5–15. Cells were incubated in AlamarBlue for 4 h, and the media were collected and the absorbance was measured at 570 and 630 nm wavelengths (Robonik, Elisa Plate Analyzer). After incubation, the media were collected and fresh growth media were added to the substrates. A baseline measurement was recorded 1 day after cell seeding and repeated at day 2, 4, 7 and 10.

### 2.4. ZnO and Si NW Degradation Cytotoxicity Assay

To evaluate the effects of toxic by-products potentially released by Si and ZnO wafers or Si and ZnO NWs grown for 1 and 3.5 h, the substrates were incubated in cell culture media up to 7 days at 37 °C (“conditioned media”). After 3 days of incubation, media were collected, and the substrates were incubated with new fresh media for another 4 days. Three different culture conditions were tested for each substrate: 1, 2 and 4 mL of medium per sample cm^2^. The conditioned media were then used as growing medium for cardiac stromal cells seeded in 96-well cell culture plates, at a density of 5 × 10^3^ cells/well. To evaluate the potential cytotoxicity of the conditioned media, an AlamarBlue assay was performed before (baseline) and 24 h after incubation in conditioned media, and the results were expressed comparing absorbance readings in conditioned medium vs. “baseline” (n = 5–10).

### 2.5. Cell Morphology

To evaluate cellular adhesion to the substrate, cell cultures were washed twice in PBS and incubated with Hoechst 33,342 nuclear staining (1 mg/mL) and a 10 µM solution of Vybrant^®^ CFDA SE dye in PBS for 15 min at 37 °C. Following incubation and removal of the staining solution, fresh cell medium was added, and representative optic fields were visually analyzed using a Leica inverted microscope (3 images for each experimental group were taken—the experiment was repeated twice). Cell area was calculated using Image J, and the results are reported as mean ± standard deviation.

### 2.6. Statistical Analysis

One-way ANOVA with Tukey modifications was used to evaluate all statistical differences among groups. Data were reported as average ± standard error of the means.

## 3. Results

### 3.1. ZnO and Si Nanowire Growth

**Figure 1:**
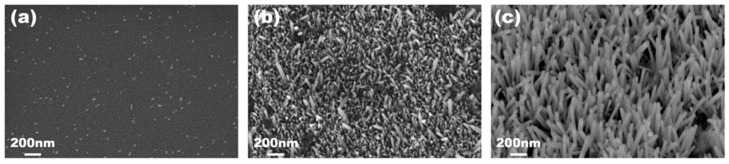
SEM images of Si samples coated with (**a**) 10-nm-thick ZnO film, and with additional wet growth of ZnO nanostructures by hydrothermal synthesis for 1 h (**b**) and 3.5 h (**c**).

The ZnO and Si NWs’ morphologies were characterized by scanning electron microscopy before cell culture. Representative images are shown in Figure 1 for ZnO NWs. The ZnO “seed layer” deposited on Si wafer (Figure 1a) shows a very smooth surface with sparse crystallites with a size of few tens of nanometers. The NW sample grown for 1 h (Figure 1b) shows elongated structures originating from the seed layer. These structures, which represent the embryonic stage of the high-aspect-ratio NW growth obtained at prolonged growth time, have an average length of 160 ± 60 nm. On the other hand, the sample grown for 3.5 h (Figure 1c) shows well-developed nanowires with an average length of 970 ± 170 nm. The NWs tend to grow preferentially along the normal direction to the substrate surface but with a significant spread in their orientation.

### 3.2. ZnO NW Biocompatibility

**Figure 2:**
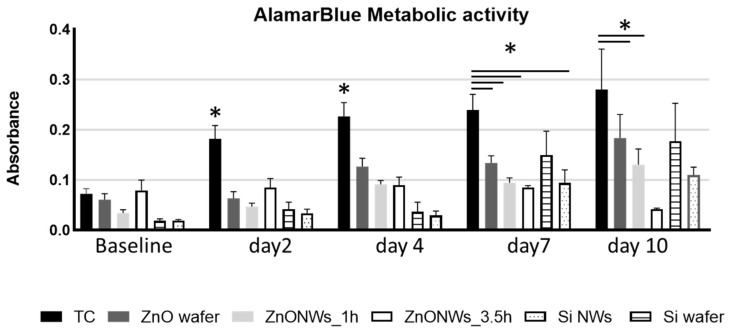
Cardiac stromal cells’ metabolic activity assay cultured in tissue culture plastic (TC), ZnO- and Si-coated wafer, and Si and ZnO nanowires. * *p* ≤ 0.05.

Cardiac stromal cells were cultured on top of Si or ZnO wafers and Si or ZnO NWs samples, and their survival and growth were evaluated by AlamarBlue metabolic activity assay. After 24 h (baseline), no significant differences were observed among CSCs cultured on top of the different substrates, although a lower absorbance in both Si wafer and NW was observed as compared to the ZnO-NW_1h group and the ZnO-NW_3.5h, which showed higher absorbance values. Similar results were observed after 2 and 4 days; the TC group showed a significant increase in the absorbance measurement compared to all other groups, which only showed a modest increase in cell growth compared to their baseline values (Figure 2). After 7 days, and up to 10 days, the TC group showed only a modest increase in cell growth, likely due to cell confluency in the plate and cell–cell contact inhibition. However, a significant increase was still observed at day 7, compared to both Si and ZnO NW groups (Figure 2) and a non-significant trend when compared to the Si wafer. Interestingly, among the five different substrates tested, both Si wafer and Si NW groups showed a more robust increase as compared to day 4, which continued until day 10 (Figure 2). On the other end, the ZnO wafer group showed a steady growth over the 10 days of analysis, although not significant, compared to the other groups. When looking into the ZnO NW groups, only the ZnO-NW_1h group showed a modest increase in cell growth up to 10 days, while the ZnO-NW_3.5h group showed comparable absorbance values up to day 7 followed by a decrease in absorbance at day 10. A summary of the absorbance values is reported in Supplementary Table S1.

**Figure 3:**
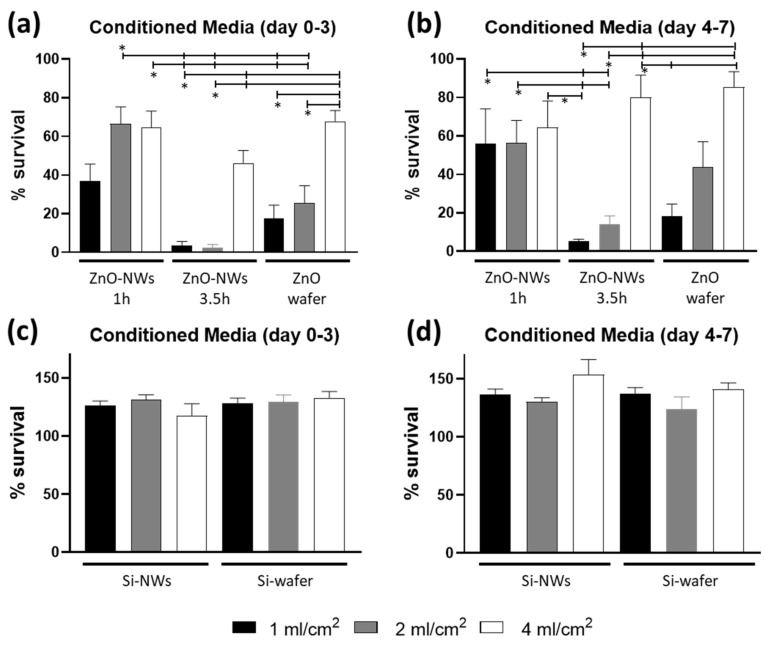
Cytotoxicity assay of ZnO- (**a**,**b**) or Si- (**c**,**d**) based substrate-conditioned media collected after 3 (**a**,**c**) and 7 days (**b**,**d**). * *p* ≤ 0.05.

We then looked into the effects on cell cultures related to the degradation products from the ZnO wafer and ZnO NW samples (Figure 3) and found that some degree of cytotoxicity was in fact evident, indicating that prolonged culturing conditions in the absence of medium refresh may affect cell survival due to the accumulation of by-products from the ZnO ultrathin layer on Si or ZnO NWs and/or for the production of some metabolic toxic by-product by the cells. Specifically, when media were collected after 3 days of incubation, the ZnO-NW_3.5h showed higher toxicity when the substrates were incubated at a concentration of 1 and 2 mL/cm^2^, showing a cell survival below 10% and significantly lower compared to the 2 mL/cm^2^ ZnO-NW_1h and all 4 mL/cm^2^ groups (Figure 3a). Similar results were obtained when media were collected after 7 days of incubation (Figure 3b). When the substrate-conditioned media volumes were larger, cell survival improved up to 50% in both conditions without relevant differences related to the incubation volumes (Figure 3). All 4 mL/cm^2^ groups showed better cell survival compared to 1 and 2 mL groups, mainly when media were collected after 3 days of incubation with the ZnO-NW_3.5h showing again a non-significant lower cell survival compared to the other groups (Figure 3a). Lastly, when media were collected after 7 days of incubation, the ZnO-NW_1h groups showed a survival around 60–70% with no differences among the three different concentrations, while the ZnO-NW_3.5h and the ZnO wafer groups showed cell survival in a concentration-dependent manner (Figure 3b). On the other end, when the Si wafer and Si NWs were incubated in cell growth media for 3 and 7 days, no signs of cytotoxicity were observed with cells continuing to grow as observed by the increase in cell number (Figure 3c,d). A summary of the cell viability data is reported in Supplementary Table S2.

We further evaluated the degradation rates of the ZnO-NW_3.5h as they showed the highest cytotoxicity among all the samples. After 10 days of incubation in cell culture media, ZnO-NW_3.5h were evaluated by SEM, showing a higher degradation rate in samples incubated in higher volumes (Supplementary Figure S1).

**Figure 4:**
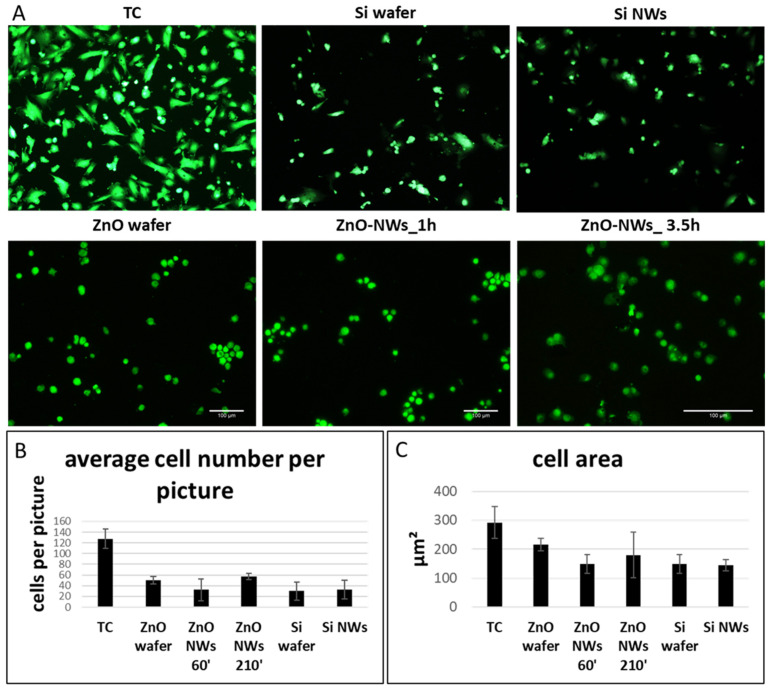
Viability assay of CSCs cultured on different Si- and ZnO-based substrates (**A**). Quantification of cell number per image (**B**) and their surface area (**C**).

We then looked into cell morphology 2 days after cell seeding. In both Si- and ZnO- based groups, cells were sparsely attached to the NW chips without a significant difference among the groups (Figure 4). The cells still retained a round morphology, indicative of poor cell adhesion to the substrate. On the other end, CSCs plated on TC plastic showed a more elongated morphology and a significantly higher cell number (Figure 4B). We then quantified the cell area, and no significant differences were observed among the groups (Figure 4C).

## 4. Discussion

We presented preliminary investigations on the use of ZnO and Si nanowires as a new approach to achieve electrical contact toward the cell membrane. Both nanostructures can be grown at low temperature, thus the growth can be achieved on the backend of electronic integrated circuits.

ZnO nanostructures are known to be biologically active as they can produce reactive oxygen species (ROS) and release Zn^2+^ ions [24]; such by-products may exert cytotoxic effects, thus the full biocompatibility of ZnO nanostructures, including NWs, is still debated [25,26]. In particular, distinct parameters (i.e., the concentration, size, shape) of ZnO nanostructures and relevant by-products can prove to be harmful, though not all cell types appear to be equally affected. Thus, for instance, on the one hand, muscular (H9c2; [27]), neuronal (NG108-15 [28]), cardiac (HL-1; [28]), fibroblastic (NIH 3T3) and umbilical-vein-derived endothelial [29,30] cell lines all show some degree of sensitivity to ZnO NWs. However, it has also been reported that small amounts of ZnO NWs can promote cell growth, proliferation, differentiation and tissue regeneration, as shown for PC12 neuronal cells [27], HeLa cells [31] and for L929 cells [32]. Furthermore, angiogenesis and osteointegration have been reported to be somehow supported by the ZnO antibacterial and antifungal properties [33,34]. Last, besides a number of biomedical, health- and sustainability-related applications [35,36]. it is worth noting that the ZnO NWs’ cytotoxicity makes them potential candidates for the targeted elimination of cancer cells [37,38]. Our work, in line with others reported elsewhere, shows some degree of cytotoxicity when cells were cultured on the ZnO NWs substrates. A recent work has shown that ZnO cytotoxicity, degradation and absorption by the cells can also be affected by serum proteins such as albumin and casein or by glucose [39]. However, in the study, the authors evaluated ZnO nanoparticles as compared to our study, which looked into the degradation of ZnO and Si NWs, and we cannot exclude that different shapes can influence protein binding to the NW and further degradation. In this regard, evaluating NWs’ degradation in H_2_O, alone or in combination with a single serum protein may be relevant to better understand the degradation potential of the NWs in respect to the culture media. Taken together, our data indicated that ZnO substrates may release toxic products when in culture for long periods and that the presence of NWs may slow down both the cell adhesion process at the beginning of the cell culture and the growth rate; nonetheless, CSCs are still able to keep growing for up to 10 days in culture on engineered substrates, thus confirming the biocompatibility of both ZnO NWs and ultrathin ZnO films on Si. As these cells are known to be important in tissue homeostasis and to be supportive for cardiomyocyte maturation and the microenvironment, our preliminary data suggest that they can be cultured, alone or in combination with other cells such as cardiomyocytes, on these devices for potential in vivo tissue construct development [40,41,42].

As for the ZnO nanowires’ synthesis, the growth technique reported in this work is a well-established wet chemical route akin to what is generally found in the literature showing cytotoxicity studies and electrical contact approaches to cells using ZnO. What we point out as a remarkable parameter is the use of very thin and short NWs, with length well below 1 μm (as also found in ref. [28]), while most of the works deal with rods and more complex structures such as nanoflowers, which are in the few-microns scale [26,27,30,31]. Certainly, short nanowires with a small diameter can ensure a more intimate contact with the cells; however, their high surface to volume ratio can be detrimental to cell viability at a higher rate of ZnO dissolution. The cytotoxicity of ZnO NWs, even though not present for all cell lines, is definitely a severe drawback and represents the main limitation for the use of such nanostructures as an electronic bio-interface. A possible paradigm for the solution of such an issue, which may drive the future research in this field, is the encapsulation of the nanostructures with properly conductive CMOS-compatible material, which can act as an inhibitor for the ZnO dissolution. As an example of feasibility of coating the ZnO NWs with encapsulating material, though with electrically insulating behavior, Lee et al. reported on ZnO NWs coated with 5-nm-thick SiO_2_, which were used to decrease the adhesion to the substrate of HUVECs and NIH 3T3 fibroblasts [43]. On the other end, Si-based substrates have shown much less concern in terms of cell cytotoxicity as Si is known to be an inert material. However, in order for these applications to be effective and successful, an excellent cell membrane–NW interaction is fundamental. The essential condition for the growth of NWs on the CMOS sensor is a dramatic reduction of the process temperatures, with respect to the commonly adopted technique, the CVD, which can easily reach 1000 °C. In this paper, we refer to a recently patented technique [25], which allows the growth of Si NWs, and silicon nanostructures (Si-ns) in a variety of different regimes, all characterized by low process temperature. The technique is based on confining the heating merely to the metal nanoparticles deposited on the substrate, obtained by irradiation with microwaves (MW). The so-called nano-susceptors, due to their very limited dimension, avoid the induction of strong currents by the electromagnetic impinging wave, thus avoiding a complete reflection of the wave. Due to the extremely reduced mass, heat can produce a large local increase in temperature. In these conditions, when the substrate is made out of silicon (as for integrated electronic circuits) or glass or plastic (namely with a conductivity many orders of magnitude lower than the metals), it does not heat up, and only the nanoparticles used as nano-susceptors can reach high temperatures. Commonly, gold is used for silicon nanowire growth, but, currently, this metal is forbidden due to the strong contamination produced. We satisfactorily used tin as a substitute for gold. The presence of a metal droplet contributes to the growth with the catalyst of the silane gas, once the eutectic temperature is overcome, giving rise to the release of silicon. The process involved is referred to as vapor–liquid–solid (VLS), with the dissolution of the silane molecule at the contact with the heated droplet, followed by the dilution of silicon in the melted metal and finally by the deposition of silicon once oversaturation is reached. By using this technique, we successfully produced substrates covered with Si NWs of different length and shape and successfully grown and tested for physiological responsiveness in different cell types (including primary neuronal and microglial cells from mice [44]). Importantly, these Si NWs proved to be biocompatible with the cells tested, showing unaffected cell morphology, membrane currents and ATP-elicited intracellular Ca^2+^ transients [45,46,47]. In our work, we showed that Si devices can be used to 2D culture CSCs and that the process to generate Si NWs is biocompatible and can potentially be integrated into 3D cardiac stromal cell constructs. As Si-based devices have shown excellent biocompatibility when tested both in vitro and in vivo [48,49], our data using a newly tested cell type are in line with previously published data. Recently, the addition of Si to classical Ti alloys has been shown to be highly biocompatible, both in vitro and in vivo [50]. Moreover, the authors also demonstrated that these newly generated alloys increased osteoblast and osteoclast activity in vivo as compared to the control group, indicative of better tissue regeneration and alloy integration [50].

A CMOS-integrated nanoelectrode has been proved to be able to simultaneously record intracellular membrane potentials from hundreds of connected in vitro neonatal rat ventricular cardiomyocytes [51]. The same group has recently developed a Si-based nanoelectrode array that consists of platinum-black electrodes with nanoscale roughness fabricated on top of a silicon chip. The authors showed that their device can be configured into the pseudo-current-clamp mode (for concurrent current injection and voltage recording) or into the pseudo-voltage-clamp mode (for concurrent voltage application and current recording) [52]. A similar approach was established by Liu et al. and generated a new platform of ultrasharp Si nanowire arrays capable of permeating, recording and stimulating intracellular activity in neuronal and cardiac networks [53]. Recently, Si NWs have been reported to improve 3D cell–cell adhesion, thus facilitating oxygen supply to the center of spheroids, and electrical conductivity, improving the tissue function of hiPSC cardiac spheroids [54]. These results lay down a solid foundation for the development of suitable nanowired hiPSC cardiac spheroids as an innovative cell delivery system to treat cardiovascular diseases. For decades, our group has been dealing with cardiac spheroids, having been the first to use them as a model of the cardiac microenvironment and to study their biotechnological potential.

In conclusion, we have shown that CSCs can be cultured on ZnO or Si NW devices and that the cells can grow for up to 10 days, although significantly slower compared to the regular TC culture condition. The data presented here represent a fundamental step for advancing the studies on the application of these devices in tissue engineering, both scaffold rich and scaffold free (spheroids), and, more generally, in regenerative medicine. However, we did not fully investigate whether their functional properties such as expression of cardiac markers or paracrine activity are maintained. Similarly, a NW coculture with cardiomyocytes also needs to be tested to evaluate the behavior of both cell types and the capability of CSCs to support the CMs’ functional properties. These studies are of paramount importance and will represent the next step for the generation of a CMOS-integrated 3D cardiac tissue.

## Data Availability

The data presented in this study are available on request from the corresponding author.

## Supplementary Materials

The following supporting information can be downloaded at: https://www.mdpi.com/article/10.3390/bioengineering9110621/s1, Figure S1: SEM images of Si samples coated with 10-nm-thick ZnO film and with additional wet growth of ZnO nanostructures by hydrothermal synthesis after 1 week incubation in (a) 0.25 mL, (b) 0.50 mL, (c) 1.0 mL, and (d) 2.0 mL cell culture media.; Table S1: Absorbance measurements of Cardiac Stromal cells grown on tissue culture plastic (TC), ZnO-NanoWires coated Si wafer (ZnO-NWs 1 h and 3.5 h), ultra-thin ZnO film (‘seed layer’) on Si wafer (ZnO wafer), Si wafer (‘seed layer’) and Si-NanoWires coated Si wafer; Table S2: Cardiac stromal cell viability 24 h after incubation with cell culture media incubated with ZnO-NanoWires coated Si wafer (ZnO-NWs 1 h and 3.5 h) or ultra-thin ZnO film on Si wafer (ZnO wafer), Si wafer (‘seed layer’) and Si-NanoWires coated Si wafer for 3 days (Table S2a) and for other 4 days (Table S2b).
