# Supplementary material for: Biocompatibility and Connectivity of Semiconductor Nanostructures for Cardiac Tissue Engineering Applications
Source: Bioengineering (Basel). 2022 Oct 27;9(11):621. doi: 10.3390/bioengineering9110621 (PMC9687646; doi:10.3390/bioengineering9110621)
Supplement: Supplementary file 1 [file bioengineering-09-00621-s001.zip › bioengineering-1975487-supplementary.pdf]

Supplementary Figure S1

SEM images of Si samples coated with 10-nm-thick ZnO film and with additional wet growth of ZnO nanostructures by hydrothermal synthesis after 1 week incubation in (a) 0.25 mL, (b) 0.50 mL, (c) 1.0 mL, and (d) 2.0 mL cell culture media.

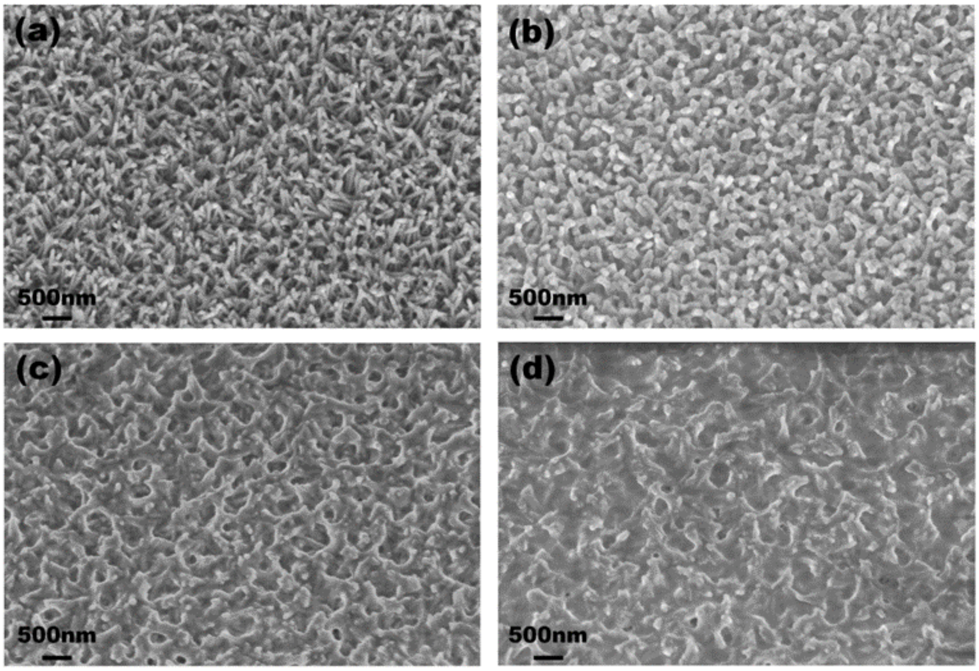

Supplementary tables

Suppl. Table S1.

Absorbance measurements of Cardiac Stromal cells grown on tissue culture plastic (TC), ZnO-NanoWires coated Si wafer (ZnO-NWs 1h and 3.5h), ultra-thin ZnO film ('seed layer') on Si wafer (ZnO wafer), Si wafer ('seed layer) and Si-NanoWires coated Si wafer.

AVERAGE						
	**days**	**baseline**	**2**	**4**	**7**	**10**
**TC**		0.067 ± 0.013	0.160 ± 0.032	0.170 ± 0.015	0.172 ± 0.018	0.205 ± 0.033
**ZnO wafer**		0.060 ± 0.011	0.063 ± 0.013	0.126 ± 0.016	0.133 ± 0.014	0.183 ± 0.047
**ZnO NWs 1h**		0.034 ± 0.006	0.046 ± 0.006	0.091 ± 0.007	0.094 ± 0.010	0.130 ± 0.031
**ZnO NWs 3.5h**		0.079 ± 0.020	0.084 ± 0.017	0.089 ± 0.016	0.085 ± 0.003	0.041 ± 0.002
**Si wafer**		0.018 ± 0.004	0.041 ± 0.014	0.036 ± 0.019	0.149 ± 0.047	0.177 ± 0.075
**Si NWs**		0.020 ± 0.002	0.042 ± 0.008	0.040 ±0.016	0.132 ± 0.032	0.109 ± 0.015

Suppl. Table S2

Cardiac stromal cell viability 24h after incubation with cell culture media incubated with ZnO-NanoWires coated Si wafer (ZnO-NWs 1h and 3.5h) or ultra-thin ZnO film on Si wafer (ZnO wafer), Si wafer ('seed layer') and Si-NanoWires coated Si wafer for 3 days (table S2a) and for other 4 days (table S2b).

Table S2a

Conditioned media days 1-3		
	**viability (%)**	**SEM**
**ZnO wafer 1 mL/cm^2**	17.61	± 6.82
**ZnO wafer 2 mL/cm^2**	25.57	± 8.96
**ZnO wafer 4 mL/cm^2**	67.88	±5.60
**ZnO-NWs 1h 1 mL/cm^2**	44.31	± 6.70
**ZnO-NWs 1h 2 mL/cm^2**	66.61	± 8.74
**ZnO-NWs 1h 4 mL/cm^2**	64.54	± 8.62
**ZnO NWs 3.5h 1 mL/cm^2**	3.37	± 2.31
**ZnO NWs 3.5h 2 mL/cm^2**	2.23	± 1.81
**ZnO NWs 3.5h 4 mL/cm^2**	46.33	± 6.41
**ZnO NWs 3.5h 4 mL/cm^2**	46.33	± 6.41
**Si wafer 1 mL/cm^2**	128.03	± 4.52
**Si wafer 2 mL/cm^2**	128.17	± 6.06
**Si wafer 4 mL/cm^2**	132.78	± 5.43
**Si NWs 1 mL/cm^2**	126.39	± 3.76
**Si NWs 2 mL/cm^2**	131.04	± 4.29
**Si NWs 4 mL/cm^2**	117.36	± 10.39

Table S2b

Conditioned media days 4-7		
	**viability (%)**	**SEM**
**ZnO wafer 1 mL/cm^2**	18.26	± 6.40
**ZnO wafer 2 mL/cm^2**	43.74	± 13.28
**ZnO wafer 4 mL/cm^2**	85.33	± 8.03
**ZnO-NWs 1h 1 mL/cm^2**	55.95	± 18.07
**ZnO-NWs 1h 2 mL/cm^2**	56.33	± 11.69
**ZnO-NWs 1h 4 mL/cm^2**	64.31	± 13.83
**ZnO NWs 3.5h 1 mL/cm^2**	5.19	± 1.12
**ZnO NWs 3.5h 2 mL/cm^2**	13.93	± 4.43
**ZnO NWs 3.5h 4 mL/cm^2**	79.92	± 11.65
**Si wafer 1 mL/cm^2**	137.25	± 5.03
**Si wafer 2 mL/cm^2**	123.82	± 10.56
**Si wafer 4 mL/cm^2**	140.99	± 5.47
**Si NWs 1 mL/cm^2**	136.14	± 4.86
**Si NWs 2 mL/cm^2**	130.25	± 3.45
**Si NWs 4 mL/cm^2**	153.45	± 14.04
